# Evaluating exposure to vehicle pollutants using physics-informed immersive reality models

**DOI:** 10.1098/rsos.241111

**Published:** 2024-09-25

**Authors:** Run Si, Jason Stafford

**Affiliations:** ^1^ School of Engineering, University of Birmingham, Birmingham B15 2TT, UK

**Keywords:** air quality, human-centric, non-exhaust emissions, pollution modelling

## Abstract

Major health risks and chronic diseases are caused by exposure to unregulated particle pollutants from road, tyre and brake sources. Here, we use large-eddy simulations to identify local exposure to these harmful pollutants and build a physics-informed immersive reality experience to communicate outcomes with the general public for health guidance. Our analysis reveals that exposure to non-exhaust pollution is greatest at the end of braking phases, when deceleration rates are above 3 m s^−2^, diminishes to background levels for pedestrians located 1.5 m away from a car, and is reasonably insensitive to the car type. We show that by using immersive reality models to visualize pollution data in a human-centric format, people could identify pollutant sources and health risks, and understand how to navigate urban spaces for reduced exposure. This was achieved without any prerequisite knowledge and with minimal dependency on educational background, suggesting the approach can support public health guidance, policymakers and urban planners towards improving air quality in urban environments.

## 1. Introduction

Around four million people died from exposure to fine and ultrafine particle matter (PM${,}_{2.5}$) in 2019, leading to the World Health Organization (WHO) updating the annual mean recommendation to 5 μg m^−3^ in 2021. Non-exhaust emissions are a prominent contributor to outdoor air quality, exist for all road vehicles [1] and can surpass exhaust emissions [2]. These particle pollutants are categorized by emissions from abrasion (brake, tyre and road surface wear) and road dust resuspension [3,4].

Previous epidemiological studies show that the effects of short-term exposure and long-term exposure can lead to several fatal outcomes [5], cardiovascular and respiratory diseases [6], and significantly increase mortality and morbidity in cities [7]. Other common health issues include chronic obstructive pulmonary disease, respiratory infections, stroke, lung cancer, ischaemic heart disease and neonatal disorders [8–10].

Preventative measures that improve health outcomes are lacking, with no regulations on vehicle manufacturers and technical challenges due to complex particle emission routes from the source to the environment [11]. Total emission factors for all road conditions from battery-electric vehicles have been suggested to be 7%–12% greater than equivalent diesel and petrol vehicles under Euro 6 standard for PM${,}_{10}$ and 1%–5% greater for PM${,}_{2.5}$, mainly attributed to the vehicle mass increase [12]. Urban areas, where most of the world’s population lives, present the biggest concern with frequent braking and close proximity between vehicles, cyclists and pedestrians. Indeed, United Nation’s projections suggest that 68% of the world’s population will live in urban areas by 2050 (today, it is 55%), meaning a large and increasing number of people are at risk of exposure and the negative consequences for health and longevity. There is a critical need, therefore, to identify exposure risks, raise awareness and develop scalable solutions that protect the health of populations from this persistent air pollution source.

In this study, we develop an open-source vehicle model to perform detailed simulations of PM${,}_{2.5}$ pollution transport from emission sources to the surrounding spaces that pedestrians and cyclists occupy. We reveal local exposure risks during urban driving by using computational fluid dynamics to simulate the spatio-temporal evolution of these pollutants from vehicles to the environment. Using this space- and time-resolved data, we construct a human-centric virtual urban environment that conveys exposure information by placing users in this virtual space, enabling them to navigate and observe pollutant emissions that are invisible in the real world. We then deployed this immersive model on the public and assessed its effectiveness for population health guidance.

## 2. Material and methods

**Figure 1.**
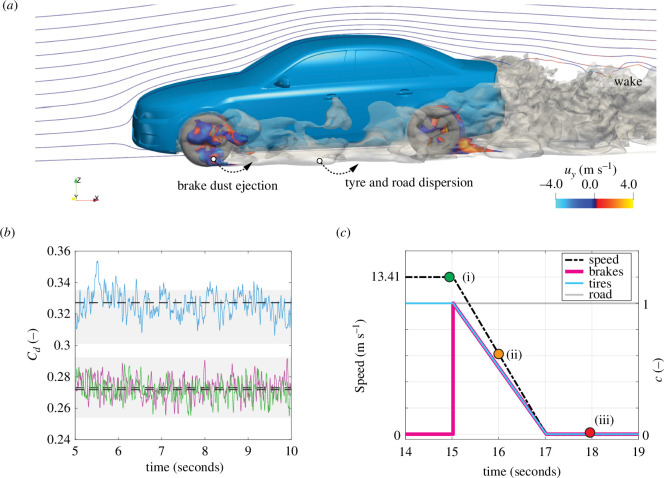
Computational modelling of vehicle non-exhaust emissions. (*a*) Airborne pollution snapshot during braking. (*b*) Computed drag coefficients for notchback (purple), fastback (green) and estateback (blue) vehicles. Dashed lines show time averages with grey zones representing spread in experimental data [13]. (*c*) Example braking profile from cruising to stopping, along with corresponding emission source profiles (c).

**Figure 2.**
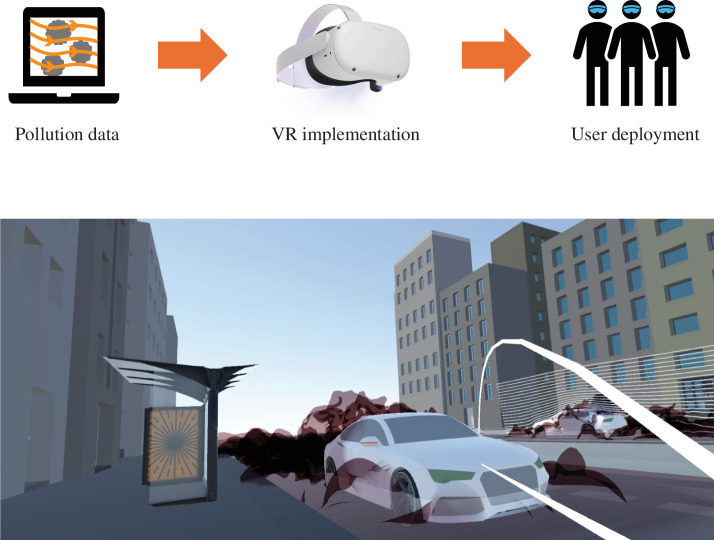
A human-centric immersive approach for visualizing vehicle pollutants in urban spaces, including the model workflow (top) and a snapshot from the virtual environment experienced by the public (bottom).

The research methods for this multi-disciplinary investigation spanned three core areas: (i) computational modelling of vehicle pollutants, (ii) development of human-centric virtual reality (VR) models for data visualization, and (iii) deployment of these immersive models for public health guidance. First, we used computational fluid dynamics to investigate the transport of non-exhaust pollutants from the source to the nearby environment (figure 1*a*). Second, we transferred the numerical predictions into VR and created a virtual urban environment for human-centric data visualization (figure 2). The third step involved deploying the VR solution to the public and analysing the outcomes from this participation to understand the effectiveness as a health guidance solution. Below, a description of each method has been provided. An extended method has also been included in the electronic supplementary material, which describes additional details of the methodologies and resources used.

### 2.1. Computational fluid dynamics

An open-source vehicle geometry traditionally used for aerodynamics investigations was adapted for the current work. The DrivAer model was originally developed to enable a wider research community to perform aerodynamic research investigations on realistic vehicles, including notchback, fastback and estateback designs [14]. With relatively minor modifications, including the addition of disc brakes front and rear, this generic vehicle geometry was chosen to explore PM_2.5_ emission pathways from sources (brake, tyre and road) to the surrounding environment (figure 1*a*).

A hybrid large-eddy simulation (LES) approach [15–17] was used to solve the governing equations for fluid flow (continuity, Navier–Stokes) and implemented within the open-source computational mechanics software OpenFOAM (v2206). This method has been suggested as suitable for capturing attached boundary layer flows, as well as separated flows and large-scale turbulent structures outside of the boundary layer [18]. To this end, we validated the LES predictions to ±8% of experimental drag coefficient data for notchback, fastback and estateback vehicle types (figure 1*b*). Further details on the mathematical model and numerical methods are provided in electronic supplementary material, §A.

The pollutants considered in this work (PM${,}_{2.5}$) consist of particles with diameters approximately 0.001 μm to less than 2.5 μm, Stokes numbers ≪0.1 and dilute concentrations ranging from approximately 1 to 100 μg m^−3^. This allowed for modelling emissions sources using passive scalar transport equations [19]. Non-exhaust sources also generate a large fraction of PM${,}_{10}$ particles [12]. In this study, we focused on the dispersion of PM${,}_{2.5}$ particles as these have the greatest impact on health issues through their ability to be absorbed through the respiratory system [7]. As the theoretical sedimentation rates for these larger particles are approximately 100 times higher, it is suggested that the pollutant dispersion predicted for public health guidance also provides safe distances from exposure to PM${,}_{10}$. To examine exposure to harmful particles (PM${,}_{2.5}$), we coupled the LES with transport equations to model brake, tyre and road sources individually and implemented speed and emission profiles to represent urban driving scenarios (figure 1*c*). To generalize our observations beyond specific cases or geographical locations, each pollutant type is modelled according to a dimensionless passive scalar that varies from 0≤c≤1. Here, the emission sources have the maximum concentration (c=1), and the background air quality is represented by the minimum concentration (c=0). Concentration fields were then analysed over the duration of the cruising and braking phases to quantify the risks of pollution exposure and reveal insights on the pollution dispersion mechanisms.

### 2.2. Virtual reality

The spatio-temporal pollution data from the LES models was post-processed using ParaView to create streamlines and pollutant clouds. This was used to reveal the extent of the non-exhaust emissions during various urban driving scenarios (e.g. cruising and braking). Graphical textures and shading were then added to the data using Blender and imported into a Unity3D application. Within Unity3D, a virtual environment was constructed, containing footpaths, roads, bus shelters, buildings and so on. A movable human actor was also added together with motion tracking. This allowed the user to experience the environment from their individual perspective and navigate around the virtual urban space (figure 2). This created a human-centric visualization of the pollution data, which was immersive and contained relatable physical objects and spaces that individuals see in reality as pedestrians or cyclists (electronic supplementary material, §B). This VR system was implemented using Meta Oculus Quest 2 VR headsets with controllers and tethered to high-performance laptops for operating the experience (electronic supplementary material, §E).

### 2.3. Deployment to the public

Birmingham City Centre (UK) was chosen to deploy the solution to the public. Birmingham is the second largest city in the UK by population, with inner city road traffic comprising approximately 80% cars according to 2023 road traffic statistics (Birmingham City Council). As a city, it is also the second largest contributor to UK PM${,}_{2.5}$ emissions from brake and tyre wear, based on data from the UK National Atmospheric Emissions Inventory. The immersive reality display was located at the Library of Birmingham, Centenary Square, over 5 days during the summer of 2023, where approximately 400 individuals engaged with the technology for approximately 5 min (electronic supplementary material, §C). These individuals were chosen randomly, and their participation was voluntary. They were not predefined or selected beforehand, which prevented the potential for participants from researching the topic of non-exhaust emissions before using the physics-informed VR experience. As a result, the outcomes could be exclusively attributed to the immersive experience. This study underwent ethical approval by the University of Birmingham’s Ethics Committee (reference no. ERN1247). From these users, 103 randomly selected participants (who were also not predefined) were asked to complete a short survey after the immersive experience (electronic supplementary material, §D1). The outcomes were then evaluated using statistical and multi-nomial logistic regression methods (electronic supplementary material, §D2) to assess the effectiveness of the physics-informed immersive approach.

### 2.4. Data, material and software availability

Our study has been performed using open-source vehicle data and computational modelling software. Links to our repositories, which contain the computational models and instructions on the implementation of the simulation results into Unity software for VR deployment, are provided in [20,21]. Further details of our models, analyses and resources are provided in the electronic supplementary material.

## 3. Results and discussion

**Figure 3.**
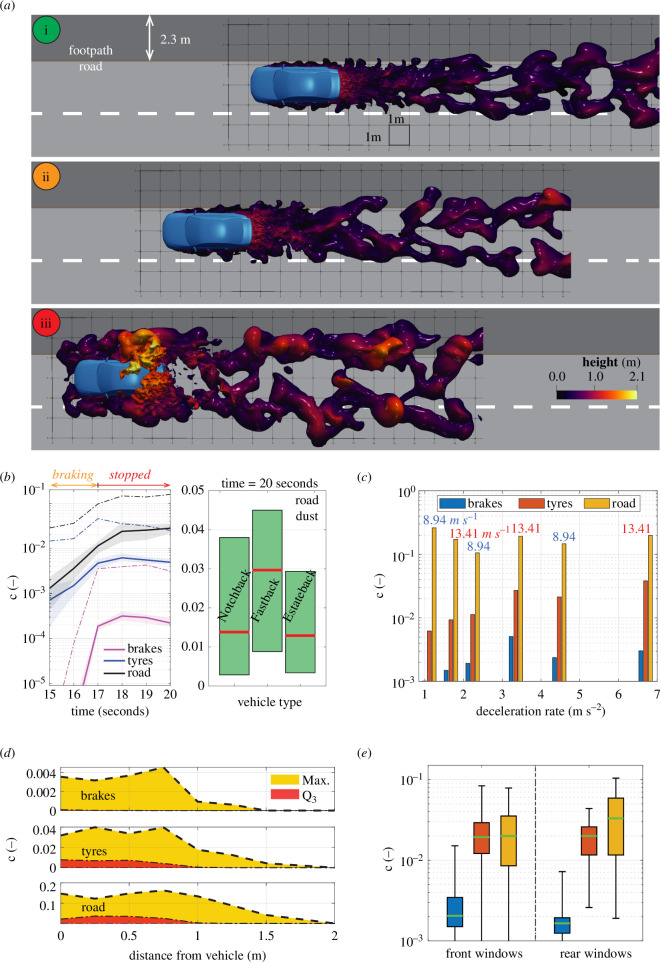
Local exposure to vehicle non-exhaust emissions in urban environments. (*a*) Proximity between pollutant plumes and pedestrian spaces. Snapshots (i–iii) correspond to points along the braking phase identified in figure 1*c*. (*b*) Effect of vehicle type on exposure. Solid lines with shaded areas show average c and vehicle variation. Dashed lines show maximum c. (*c*) Effect of deceleration rate and car speed on exposure. (*d*) Maximum and inter-quartile (Q_3_) concentration exposure levels for pedestrians and cyclists. (*e*) Exposure risks to vehicle passengers with open windows (same legend as part (*c*)).

We first investigated exposure risks for pedestrians using computational models to simulate the turbulent flow of non-exhaust pollution from sources to the environment (figure 1*a*). When a vehicle decelerates from urban cruising speeds, these pollutants extend into pedestrian spaces by approximately 2 m and simultaneously rise to heights of approximately 2 m above ground (figure 3*a*), easily enveloping cyclists and pedestrians. This behaviour is driven by the formation of an adverse pressure gradient, flow separation and reversal on the vehicle body during the vehicle deceleration phase, which pulls the wake forwards and spreads pollutants upwards and outwards. We analysed the pollution concentration 100 mm away from the vehicle and averaged along a 20 m road distance (0.25 m ahead to 15 m behind the car). We chose a height of 1 m off the ground to consider the most at-risk groups of the elderly and children under the age of 5 years (based on WHO global height data). Notably, the highest exposure to pollutants occurs when the vehicle stops (figure 3*b*), indicating that the greatest risk is where stopping traffic and pedestrian gatherings coexist (e.g. pedestrian crossings, traffic signals and bus stops).

Interestingly, the effects of vehicle type on average exposure during the braking and stopped phases are not substantial, with moderate differences between the median and interquartile ranges observed for road dust emissions (figure 3*b*). This allowed us to focus our analysis on a single vehicle type, which we chose as the notchback geometry (figure 1*a*). We tested variations in cruising speed (8.94–13.41 m s^−1^, equivalent to 20–30 mph) and deceleration rate (approx. 1–7 m s^−2^) by examining ranges observed in real braking scenarios in urban areas [22]. Reducing vehicle speed from 13.41 to 8.94 m s^−1^ has negligible effect; however, exposure to brake and tyre emissions is lowered when deceleration rates are less than 3 m s^−2^, reducing concentrations by twofold to fivefold (figure 3*c*). This highlights the importance driving behaviour may have on pedestrian exposure.

Investigating the worst-case scenario of maximum deceleration rate (6.7 m s^−2^), and after the vehicle has come to a stop (figure 3*a*(iii)), we identify a minimum safe distance of 1.5 m from the vehicle (figure 3*d*). Concentration data were analysed along the footpath, and from this, the inter-quartile (Q${,}_{3}$) was obtained, showing a drop-off in pollution exposure at approximately 1 m. This indicates that 75% of pedestrian positions along the footpath, which are 1 m from the vehicle, have low exposure to PM${,}_{2.5}$ from passing vehicles (c≈0, or background levels). However, the maximum concentrations (‘Max’) were also analysed to determine the peak pollution exposure level that can occur due to the highly stochastic, turbulent particle dispersion. At 1.5 m from the vehicle, we observe a complete reduction in brake particulates and significant reductions in tyre and road particulates. Beyond this, we find that pedestrians are statistically unlikely to be exposed to non-exhaust pollutants from adjacently passing vehicles. Note, however, that exposure to the background pollution levels remains, which is typically higher near roadways [23]. Furthermore, as the pollutants also envelope the vehicle body at the end of the braking phase (figure 3*a*(iii)), open windows can lead to passenger exposure at concentration levels similar to that of nearby pedestrians (figure 3*e*).

Next, we implemented the simulation data within a virtual environment, facilitating human-centric visualization of vehicle pollutants (figure 2). This approach accounted for user height when wearing the VR headset, giving individuals a true sense of their proximity to objects and pollutants while navigating the urban space. We deployed it in Birmingham City Centre (UK) and gathered survey responses from 103 public participants (see electronic supplementary material). We then assessed the population response using multi-nomial logistical regression analysis on the following variables: education level, sustainability level, knowledge of non-exhaust emissions beforehand, and interest towards VR technology.

**Figure 4.**
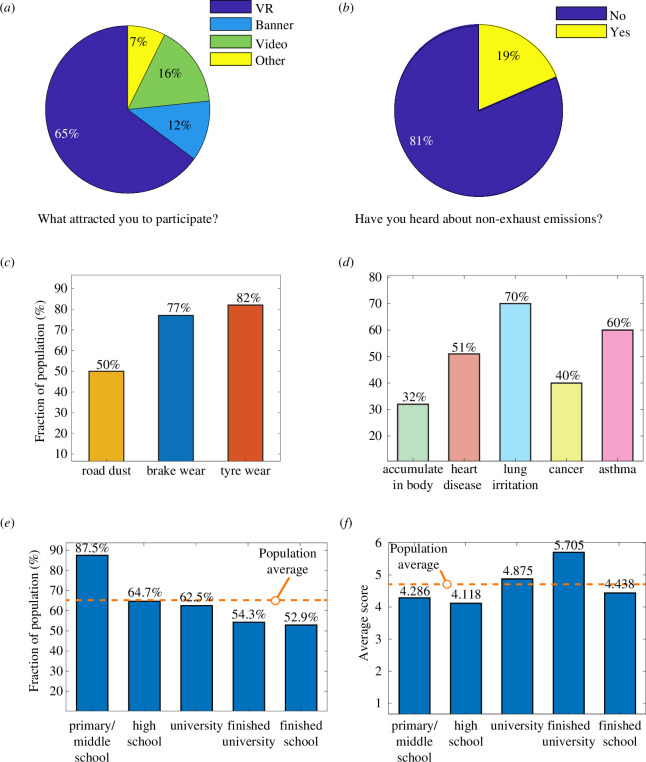
Effectiveness of the human-centric immersive approach. (*a*) Information formats which attracted members of the public to engage. (*b*) Prior awareness of non-exhaust emissions among the sample population. (*c*) Fraction of the public who responded correctly to identify non-exhaust emissions and (*d*) health issues associated with fine particle emissions. (*e*) Interest in VR approach and (*f*) average response score based on education level.

We found that 81% of people are unaware of non-exhaust emissions, emphasizing the need to improve engagement (figure 4*b*). After participating in the immersive experience for 5 min, most people recalled the composition of pollutants (figure 4*c*) and many of the associated health issues from exposure (figure 4*d*). Educational background provided a proxy to explore technology engagement and learning outcomes. An anticipated trend emerged with younger primary and middle school participants demonstrating the highest interest in VR (figure 4*d*). Although lower than the average (65%), most of the group who have finished education (52.9%) were also attracted by the VR delivery method over other information modes such as video and poster banner displays (figure 4*a*).

Considering all response data, we find that education level marginally influences participant scores on the composition of pollutants and their effects on health (figure 4*e*). The regression analysis reveals only two variables with a statistically significant correlation (p<0.05). The higher the education level, the less likely the individual will score low (β=−1.52; p=0.049), and the more sustainable a person’s lifestyle, the more likely they will score high (β=1.31; p=0.043). Removing the primary and middle school group due to a lower sample size (N=7), no variables correlated with learning performance (p>0.05). These findings suggest that the approach fosters broadly positive outcomes independent of an individual’s knowledge or educational background.

## 4. Conclusion

This study presents a physics-based immersive reality approach and examines the feasibility of engaging the public on air quality challenges and providing public health guidance. We identified localized pollutant exposure risks to persistent non-exhaust emissions in urban environments, explaining the underlying physical mechanisms and vehicle parameters that lead to increased exposure for pedestrians. Notably, the effect of vehicle aerodynamics on the transport of PM${,}_{2.5}$ particulate matter towards pedestrians and cyclists has been elucidated during several braking conditions from 1 to 7 m s^−2^. Exposure risk was highest at the end of braking events and diminished to background levels when the distances were greater than 1.5 m. However, most bus stops, pedestrian crossings and cycle lanes are within these distances and often located at the braking zones of cars (e.g. road junctions) where the largest pollution dispersion distances are found (greater than 2 m). These outcomes highlight the air quality issues with current layouts, while also supporting the redesign and navigation of urban spaces for cleaner air, particularly in situations where vehicle traffic is unavoidably close to pedestrians and cyclists. This can be aided by our observations that variation in car type (sedan, fastback and estate) is not a dominant factor. Our research also suggests that translating pollution data into human-centric immersive experiences is both an effective and agnostic solution for delivering health guidance to society. This was demonstrated by weak correlations between learning outcomes, education level and pre-existing knowledge. The synergies observed with sustainability suggest that the promotion of sustainable lifestyles will positively impact the effectiveness of this approach for clean air challenges. Complementary investigations to understand if there are two-way benefits would be valuable and may promote wider ecological health and net-zero aims. Finally, while this feasibility study focuses on non-exhaust emissions, it is applicable to all pollution challenges where there are similar needs to engage multiple stakeholders, communicate outcomes from complex physico-chemical models or experiments and enact positive societal change.

## Data Availability

Our study has been performed using open-source vehicle data and computational modelling software. Links to our repositories, which contain the computational models and instructions on the implementation of the simulation results into Unity software for VR deployment, are provided in [20,21]. Further details of our models, analyses and resources are also provided in the electronic supplementary material [24].
